# 

**Published:** 1923-10

**Authors:** 


A JOURNAL OF THE MEDICAL SCIENCES FOR THE
WEST OF ENGLAND AND SOUTH WALES.

*PUBLISHED UNDER THE AUSPICES OF*
*THE BRISTOL MEDICO-CHIRURGICAL SOCIETY.*

EDITOR:
P. WATSON-WILLIAMS, M.D.

WITH WHOM ARE ASSOCIATED
JAMES SWAIN, C.B., C.B.E., M.S., M.D., J. LACY FIRTH, M.S., M.D.,
CAREY COOMBS, M.D.

J. A. NIXON, C.M.G., M.D., Assistant Editor.

EDITORIAL SECRETARY:
A. L. FLEMMING, M.B., Ch.B.

---

"Scire est nescire, nisi id me

Scire alius sciret."

---

VOL. XL.

BRISTOL: J. W. ARROWSMITH LTD.
London: J. W. Arrowsmith (London) Ltd.
1923.

### INDEX.

Adenoid operations, Unsuccessful—A. J. M. Wright, 84.
Altitudes, Problems of living at, 170.
Amentia, 101.
Arts, Medicine and the fine—Dr. J. A. Nixon, 1.

Baillie, Miss A. B., Resignation of, 167.
**Books, Reviews of**—
Anæsthetics, Practical—C. F. Hadfield, 208.
Anæsthetics—Sir F. W. Hewitt, 159.
Anæsthetics—Dr. J. Blomfield, 61.
Arderne's De arte phisicali, 158.
Artificial limbs—M. Little, 106.
Ashby and Wright—Diseases of children, 63.
Bacteriology, Dental—F. St. J. Steadman, 112.
Ballance, Sir C. A.—History of surgery of the brain, 63.
Balme, H.—China and modern medicine, 58.
Beesly and Johnston—Surgical anatomy, 157.
Bennett, Dr. R. A.—Suggestion and common sense, 161.
Blomfield, J.—Anæsthetics, 61.
Blood transfusion—Dr. G. Keynes, 65.
Brain surgery, History of—Sir C. A. Ballance, 63.
Brand, Dr. A. T.—Cancer, 109.
British Journal of Surgery, 169.
Brown, Dr. W. Langdon—The sympathetic nervous system, 163.
Cancer—Dr. A. T. Brand, 109.
Chest, Physical examination of the—Dr. J. Crocket, 106.
Children, Diseases of—Ashby and Wright, 63.
Children, Diseases of—Dr. B. Myers, 108.
Children, Surgical diseases of—F. C. Pybus, 162.
China and modern medicine—H. Balme, 58.
Choyce, C. C.—System of surgery, 207.
Clarkson, E. R. T.—The venereal clinic, 60.
Cobb, Dr. I. G.—Aids to organotherapy, 110.
Core, Dr. D. E.—Functional nervous disorders, 62.
Crocket, Dr. J.—Physical examination of the chest, 106.
Damaye, Dr. H.—Le médecin devant l'assistance et l'enseignement psychiatriques, 109.
Foods, Analyses of—Dr. R. H. A. Plimmer, 65.
Fowler, Sir J. K.—Problems in tuberculosis 206.

**Books, Reviews of** (*continued*)—
General practice, Index to—Dr. A. C. Stark, 207.
Gordon, Dr. A. K.—Hæmatology, 204.
Greffes testiculaires—Dr. S. Voronoff, 159.
Groves, E. W. H.—Synopsis of surgery, 163.
Hadfield, C. F.—Practical anæsthetics, 208.
Hæmatology—Dr. A. K. Gordon, 204.
Hæmorrhoids—A. S. Morley, 206.
Heart as a power chamber, The—Dr. H. Sainsbury, 110.
Hernia—J. Hutchinson, 205.
Hewitt's Anæsthetics, 159.
History of brain surgery—Sir C. A. Ballance, 63.
Hutchinson, J.—Hernia, 205.
Jones and Lovett—Orthopedic surgery, 208.
Keynes, Dr. G.—Blood transfusion, 65.
Laennec, Selected passages from, 160.
Laurens, Dr. G.—Oto-rhino-laryngology, 110.
Lejars, F.—Urgent surgery, 210.
Little, M.—Artificial limbs, 106.
Lovett, Jones and—Orthopedic surgery, 208.
McKay, Dr. W. J. S.—Lawson Tait, 164.
Medicine, Dictionary of practical—Sir M. Morris, 62.
Medicine, Text-Book of—Dr. F. W. Price, 107.
Monrad-Krohn, Dr. G. H.—Clinical examination of the nervous system, 205.
Morley, A. S.—Hæmorrhoids, 206.
Morris, Sir M.—Dictionary of practical medicine, 62.
Murrell, Dr. W.—Poisoning, 64.
Myers, Dr. B.—Diseases of children, 108.
Nauheim treatment—Dr. L. T. Thorne, 210.
Nervous disorders, Functional—Dr. D. E. Core, 62.
Nervous system, Clinical examination of—Dr. G. Monrad-Krohn, 205.
Organotherapy, Aids to—Dr. I. G. Cobb, 110.
Orthopedic surgery—Jones and Lovett, 208.
Oto-rhino-laryngology—Dr. G. Laurens, 110.
Pharmacy—Sir W. Whitla, 157.
Physiology, The new—Dr. A. R. Short, 161.
Plimmer, Dr. R. H. A.—Analyses of foods, 65.
Poisoning—Dr. W. Murrell, 64.
Practical medicine series, 110.
Pregnancy, Tumours complicating—Dr. H. R. Spencer, 164.
Price, Dr. F. W.—Practice of medicine, 107.
Prognosis, Index of—Dr. A. R. Short, 204.
Psychiatriques, Le médecin devant l'assistance et l'enseignement—Dr. H. Damaye, 109.

**Books, Reviews of** (*continued*)—
Pybus, F. C.—Surgical diseases of children, 162.
Sainsbury, Dr. H.—The heart as a power chamber, 110.
St. Andrew's Institute reports, 157.
Short, Dr. A. R.—Index of prognosis, 204 : The new physiology, 161.
Spencer, Dr. H. R.—Tumours complicating pregnancy, 164.
Stark, Dr. A. C.—Index to general practice, 207.
Steadman, F. St. J.—Dental bacteriology, 112.
Suggestion and common sense—Dr. R. A. Bennett, 161.
Surgical anatomy—Beesly and Johnston, 157.
Surgical don'ts—C. H. Whiteford, 205.
Surgery, Synopsis of—E. W. H. Groves, 163.
Surgery, System of—C. C. Choyce, 207.
Surgery, Urgent—F. Lejars, 210.
Sutton, J. B.—Tumours, 59.
Sydenham's works, 111.
Sympathetic nervous system—Dr. W. Langdcn Brown, 163.
Syphilis, Atlas of—L. v. Zumbusch, 108.
Tait, Lawson—W. J. S. McKay, 164.
Tainan hospital medical report, 160.
Thorne, Dr. L. T.—Nauheim treatment, 210.
Tuberculosis, Problems in—Sir J. K. Fowler, 206.
Tumours—J. B. Sutton, 59.
Venereal disease—Dr. G. Walker, 165.
Venereal clinic, The—E. R. T. Clarkson, 60.
Voronoff, Dr. S.—Greffes testiculaires, 159.
Walker, Dr. G.—Venereal disease, 165.
Whiteford, C. H.—Surgical don'ts, 205.
Whitla, Sir W.—Pharmacy, 157.
Zumbusch, L. v.—Atlas of syphilis, 108.

Bristol Medico-Chirurgical Society, 67, 170.
Buckmaster, Dr. G. A.—Reaction of the liquids of the body by indicators, 175.

Carwardine, T.—The diagnosis of peptic ulcer, 71.
Chemistry and medicine, The relation between—Prof. F. Francis, 119.
Clarke, Dr. C.—The physiology of diet and hygiene at the North Pole, 52.
Cortex, Injuries to the head illustrating functions of the—Dr. R. G. Gordon, 139.
Cummins, Col. S. L.—Acquired resistance to tuberculosis, 41.

Deglutition, Disorders of—Dr. J. P. I. Harty, 195.
Diabetes mellitus and insulin—Dr. A. T. Todd, 182.
Diet and hygiene at the North Pole —Dr. C. Clarke, 52.

Editorial notes, 66, 113, 167, 212.
Ether anæsthesia, Light—A. L. Flemming, 88.
Everest expedition, 170.

Fawcett, Prof. E., elected F.R.S., 167.
Feeble-mindedness, 101.
Flemming, A. L. — Light ether anæsthesia, 88.
Fox (Long) lecture, The, 119.
Francis, Prof. F.—The relation between chemistry and medicine, 119.
Fraser, Forbes—Surgical treatment of infections of the peritoneum, 29 67.

Gordon, Dr. R. G.—Injuries to the head illustrating functions of the cortex, 139.

Harty, Dr. J. P. I.—Disorders of deglutition, 195.
Head injuries illustrating functions of the cortex—Dr. R. G. Gordon, 139.
Hoarseness — Dr. E. Watson-Williams, 153.
Holborow, Dr. E. R., Obituary notice of, 214.

Iles, Dr. A. E. — Intracranial tumours and the occurrence of papillœdema, 94.
Indicators, Reaction of body fluids by—Dr. G. A. Buckmaster, 175.
Insulin in treatment of diabetes—Dr. A. T. Todd, 182.
Intracranial tumours and papillœdema—Dr. A. E. Iles, 94.

*Lancet* centenary, 212.
Library of the Bristol Medico-Chirurgical Society, 115.
Local medical notes, 70, 117, 174.

Medicine and the fine arts—Dr. J. A. Nixon, 1.

Nixon, Dr. J. A.—The debt of medicine to the fine arts, 1.
North Pole, Physiology of diet and hygiene at—Dr. C. Clarke, 52.

Obituary, 69, 214.

Papillœdema, Intracranial tumours and—A. E. Iles, 94.

Peptic ulcer, Diagnosis of — T. Carwardine, 71.

Peritoneum, Surgical treatment of infections of the—F. Fraser, 29, 67.
Psychiatry, Report on—Dr. J. O. Symes, 101.

Rattray, Dr. J. M., Obituary notice of, 69.
Reaction of body liquids by indicators—Dr. G. A. Buckmaster, 175.
Research, Opportunities for, 113.

St. Luke's day service, 213.
Society, Bristol Medico-Chirurgical 67, 170.
Somervell, T. H. — Lecture on Mount Everest, 170.

Symes, Dr. J. O.—Report on psychiatry, 101.

Todd, Dr. A. T.—Diagnosis and treatment of diabetes mellitus by insulin, 182.
Tonsil operations, Unsuccessful—A. J. M. Wright, 84.
Tuberculosis, Acquired resistance to—Col. S. L. Cummins, 41.

Ulcer, Diagnosis of peptic — T. Carwardine, 71.

Watson-Williams, Dr. E.—Hoarseness, 153.
Wright, A. J. M.—Unsuccessful tonsil and adenoid operations, 84.

J. W. Arrowsmith Ltd. Publishers Bristol

### CONTENTS OF VOLUME XL.

Page

The Debt of Medicine to the Fine Arts. By J. A. Nixon, C.M.G., M.D. Cantab., F.R.C.P. .. .. .. .. .. .. 1

The Principles of Surgical Treatment of Infections of the Peritoneum. By Forbes Fraser, C.B.E., F.R.C.S. .. .. .. .. 29

Acquired Resistance to Tuberculosis: a Factor in Clinical Type and Prognosis. By S. Lyle Cummins, C.B., C.M.G., M.D. .. 41

The Diagnosis of Peptic Ulcer and its Bearings on Treatment. By T. Carwardine, M.S. .. .. .. .. .. .. 71

Unsuccessful Tonsil and Adenoid Operations. By A. J. M. Wright, M.B., F.R.C.S. .. .. .. .. .. .. .. 84

Light Ether Anæsthesia. By A. L. Flemming, M.B., B.Ch... .. 88

Intracranial Tumours and the Occurrence of Papillœdema. By A. E. Iles, O.B.E., M.B. Lond., F.R.C.S. .. .. .. .. 94

The Long Fox Lecture: The Relation between Chemistry and Medicine. By Professor F. Francis, D.Sc. .. .. .. 119

Injuries to the Head Illustrating Functions of the Cortex. By R. G. Gordon, M.D., M.R.C.P. Edin. .. .. .. .. .. 139

Hoarseness: The Importance of Early Laryngoscopy. By E. Watson-Williams, M.C., B.A., M.B. Cantab., Ch.M. Bristol, F.R.C.S.E. .. .. .. .. .. .. .. .. 153

A Film Method for Determining the Reaction of the Liquids of the Body by Indicators. By George A. Buckmaster, M.A., M.D. Oxon. .. .. .. .. .. .. .. .. 175

On the Diagnosis and Treatment of Diabetes Mellitus, with Special Reference to the Usages of Insulin. By A. T. Todd, M.B., Ch.B. Edin., M.R.C.P. Lond. .. .. .. .. .. 182

Disorders of Deglutition. By J. P. I. Harty, B.A., M.B., B.Ch., F.R.C.S. .. .. .. .. .. .. .. .. 195

Progress of the Medical Sciences .. .. .. .. .. 101

Critical Review .. .. .. .. .. .. .. .. 52

Reviews of Books .. .. .. .. 58, 106, 157, 204

Editorial Notes .. .. .. .. .. 66, 113, 167, 212

The Library of the Bristol Medico-Chirurgical Society .. 115

Meetings of Societies .. .. .. .. .. .. .. 67, 170

Obituary .. .. .. .. .. .. .. .. .. 69, 214

Local Medical Notes .. .. .. .. .. 70, 117, 174

